# Experimental data on the development of “Post-installed Screw Pullout test” for in-situ investigation of concrete strength

**DOI:** 10.1016/j.dib.2020.106190

**Published:** 2020-08-22

**Authors:** Shah Nur Alam Sourav, Salam Al-Sabah, Ciaran McNally

**Affiliations:** a School of Civil Engineering, University College Dublin, Belfield, Dublin 4, Ireland; b Arup, 50 Ringsend Rd, Dublin D04 T6 × 0, Ireland

**Keywords:** In-situ test, Concrete strength, Post-installed screw, Test configuration, Load-displacement curve

## Abstract

This data article describes the datasets obtained during the development phase of “Post-installed Screw Pullout (PSP) test”, a new in-situ test for concrete strength assessment. The datasets presented focus on the results obtained in mortar samples. The presented PSP test method is based on a post-installed screw being pulled from the parent material. To implement this, the screws installed in mortar sample were loaded to failure and the responses were recorded in the form of load-displacement curves. The samples were tested for two types of screw under different test conditions, with variables that included hole depth, hole diameter and mortar strength. These datasets are associated with the article “The Post-installed Screw Pullout Test: Development of a Method for Assessing In-situ Concrete Compressive Strength” (Al-Sabah et al., 2020).

## Specifications Table

**Table:** 

Subject	Civil and Structural Engineering
Specific subject area	Non-destructive testing of concrete
Type of data	Tables; graphs
How data were acquired	The data relates to the load resistance and displacement as a screw is pulled from mortar using an Instron 1274 servo-hydraulic frame.
Data format	Raw, and processed (formulation of load-displacement curves using the raw data obtained)
Parameters for data collection	Screw diameter, diameter and depth of the hole, compressive strength of mortar
Description of data collection	After the installation of the screw in the predrilled hole, the screw is pulled at a constant rate of displacement of 0.10 mm/s using an Instron 1274 servo-hydraulic frame. The sample was held within a purpose-built steel frame. The load and displacement were continuously measured and recorded at a sampling rate of 100 Hz. Two LVDTs were used to obtain the displacement of the screw head relative to the sample surface. Excel 2013 was used to generate a load-displacement curve and other data of interest from the obtained raw data.
Data source location	School of Civil Engineering, University College Dublin
Data accessibility	With the article
Related research article	Salam Al-Sabah, Shah Nur Alam Sourav, Ciaran McNally, The Post-installed Screw Pull-out Test: Development of a Method for Assessing In-situ Concrete Compressive Strength, Journal of Building Engineering. In Press

## Value of the Data

• The datasets provide an understanding of the screw thread interaction with the surrounding mortar under different test conditions, which led to the selection of a suitable test configuration of the PSP test.
• As the PSP test aims to assess the in-situ concrete strength, the datasets will be of interest to researchers and engineers working in the field of in-situ evaluation of concrete structures. This research data would also be of interest to anchor manufacturers as this test method represents a possible new application area for screw anchors.
• The datasets assist in providing reproducible research details that can be useful for future development of the test method by fine tuning different aspects of the methodology adopted here.

## 1 Data Description

The research focused on the use of HUS 3H – 8 and HUS 3H – 10 screws. Commercially they are regarded as 8 mm and 10 mm screws respectively. In this article, the datasets obtained in different phases of research are presented. This includes the charts corresponding to the tests conducted as well as summary tables.

Table 1 shows the results of the investigation on the failure mode in mortar during the screw pull-out operation. The influence of presence of reaction ring around the screw was investigated and mode of failure was reported.

**Table 1:** Investigation on the failure mode during the screw pull-out.

Sample number	Sample type	Diameter, (mm)	Depth (mm)	Reaction ring	Failure type
1	Mortar	9.0	60	No	Splitting
2	Mortar	9.0	60	No	Pull-out
3	Mortar	8.5	60	No	Splitting
4	Mortar	9.0	60	Yes	Pull-out
5	Mortar	9.0	60	Yes	Pull-out
6	Mortar	9.5	60	Yes	Pull-out
7	Mortar	9.5	60	Yes	Pull-out

For the next phase a testing programme was developed that included key parameters such as the screw diameter, depth and diameter of the drilled hole and compressive strength of mortar. In total 17 separate test set-ups are chosen (numbered 1–17) and these are presented in Table 2 along with a summary of the test result (peak load). The load-displacement curves obtained in this stage of the investigation are shown in Figs. 1–17. The notation used for the sample designation in the load –displacement curve is as follows:

**Table 2:** Investigation on configuration of the PSP test.

Screw	Diameter (mm)	Compressive strength (MPa)	Test up	Depth (mm)	Designation	Peak load (kN)
HUS 3-H8	8.5	45.06	1	60	M-60-8.5-19	18.51 (splitting)
					M-60-8.5-20	*
			2	70	M-70-8.5-25	21.06
					M-70-8.5-26	19.98 (splitting)
	9.0	45.06	3	60	M-60-9.0-11	15.13
					M-60-9.0-12	12.42
					M-60-9.0-21	14.97
					M-60-9.0-22	13.91
		62.75	4	60	M-60-9.0-31	16.80
					M-60-9.0-32	16.32
					M-60-9.0-33	15.93
					M-60-9.0-34	15.23
		45.06	5	70	M-70-9.0-27	20.07
					M-70-9.0-28	18.83
		40.20	6	70	M-70-9.0-35	12.51
					M-70-9.0-36	10.79
					M-70-9.0-37	13.09
					M-70-9.0-38	12.04
		12.88	7	70	M-70-9.0-39	7.02
					M-70-9.0-40	7.55
					M-70-9.0-41	7.30
					M-70-9.0-42	5.28
		34.96	8	70	M-70-9.0-43	12.30
					M-70-9.0-44	10.67
					M-70-9.0-45	13.89
					M-70-9.0-46	11.53
		16.66	9	70	M-70-9.0-47	7.43
					M-70-9.0-48	10.04
					M-70-9.0-49	11.17
					M-70-9.0-50	7.76
	9.5	45.06	10	60	M-60-9.5-13	3.68
					M-60-9.5-14	3.20
					M-60-9.5-23	3.48
					M-60-9.5-24	3.40
		45.06	11	70	M-70-9.5-29	5.35
					M-70-9.5-30	5.39
HUS 3-H10	11.0	48.83	12	60	M-60-11.0-76	11.16
					M-60-11.0-80	14.01
		57.83	13	60	M-60-11.0-83	11.93
					M-60-11.0-85	15.17
					M-60-11.0-86	16.75
					M-60-11.0-87	15.46
					M-60-11.0-88	15.52
		58.34	14	70	M-70-11.0-78	12.40
					M-70-11.0-82	16.35
					M-70-11.0-84	16.11
		56.83	15	70	M-70-11.0-89	20.52
					M-70-11.0-90	23.07
					M-70-11.0-91	23.06
					M-70-11.0-92	17.94
	11.5	48.83	16	60	M-60-11.5-75	3.67
					M-60-11.5-79	4.12
		58.34	17	70	M-70-11.5-77	4.42
					M-70-11.5-81	4.53

⁎ Test was not completed due to the splitting failure of sample.



The specific results files include:

• Fig. 1 contains the data for tests involving mortar with an 8mm screw, a hole depth of 60mm and a hole diameter of 8.5mm;
• Fig. 2 contains the data for tests involving mortar with an 8mm screw, a hole depth of 70mm and a hole diameter of 8.5mm;
• Fig. 3 contains the data for tests involving mortar with an 8mm screw, a hole depth of 60mm and a hole diameter of 9.0mm;
• Fig. 4 repeats the testing in Fig. 4, but with a different mortar strength (62.75 MPa);
• Fig. 5 contains the data for tests involving mortar with an 8mm screw, a hole depth of 70mm and a hole diameter of 9.0mm;
• Fig. 6 repeats the testing in Fig. 4, but with a different mortar strength (40.20 MPa);
• Fig. 7 repeats the testing in Fig. 4, but with a different mortar strength; (12.88 MPa)
• Fig. 8 repeats the testing in Fig. 4, but with a different mortar strength (34.96 MPa);
• Fig. 9 repeats the testing in Fig. 4, but with a different mortar strength (16.66 MPa);
• Fig. 10 contains the data for tests involving mortar with an 8mm screw, a hole depth of 60mm and a hole diameter of 9.5mm;
• Fig. 11 contains the data for tests involving mortar with an 8mm screw, a hole depth of 70mm and a hole diameter of 9.5mm;
• Fig. 12 contains the data for tests involving mortar with a 10mm screw, a hole depth of 60mm and a hole diameter of 11.0 mm;
• Fig. 13 repeats the testing in Fig. 13, but with a different mortar strength (57.83 MPa);
• Fig. 14 contains the data for tests involving mortar with a 10mm screw, a hole depth of 70mm and a hole diameter of 11.0mm;
• Fig. 15 repeats the testing in Fig. 15, but with a different mortar strength (56.83 MPa);
• Fig. 16 contains the data for tests involving mortar with a 10mm screw, a hole depth of 60mm and a hole diameter of 11.5 mm;
• Fig. 17 contains the data for tests involving mortar with a 10mm screw, a hole depth of 70mm and a hole diameter of 11.5mm.

Table 3 shows the mix design used for the preparation of mortar sample in the investigation of the influence of compressive strength on the peak load of the PSP test. Table 4 shows the associated test results. The load-displacement curves obtained are shown in Figs. 18–25. The notation used for the sample designation in the load –displacement curve is as follows;

**Table 3:** Mix proportions for the investigation of the influence of compressive strength on pullout load.

Mix	Water-cement ratio	Cement content (kg/m^3^)	Sand-cement ratio	Age of testing (days)	Curing
Mix 1	0.50	450	3	7/14/28/100	Water
Mix 2	0.60	450	3	7/14/28/100	Water

**Table 4:** Test results for the investigation of the influence of compressive strength on pullout load.

Mix	Day of testing	Max load (kN)	Displacement at peak load (mm)	Compressive strength (MPa)
Mix 1	7	13.89	4.22	36.09
		15.55	4.15	35.24
		12.57	4.84	33.58
		13.19	4.80	
	14	16.64	5.42	45.87
		17.32	5.55	44.02
		17.18	6.47	44.38
		12.87	5.10	
	28	14.43	4.82	50.09
		17.4	5.14	48.23
		13.91	5.01	50.75
		17.11	5.68	
	100	21.89	5.94	57.79
		21.18	6.04	61.89
		20.76	5.99	56.77
		
Mix 2	7	11.6	4.84	28.71
		12.01	3.71	28.45
		12.67	4.74	28.00
		11.85	4.22	
	14	13.31	4.23	34.10
		13.99	4.79	34.99
		14.27	4.76	35.40
		12.23	4.36	
	28	13.46	4.50	40.68
		15.54	5.55	41.85
		13.57	4.93	41.19
		14.27	4.99	
	100	17.08	5.68	49.55
		17.87	5.22	51.21
		17.25	5.34	50.36
		16.49	5.11	



The specific results files include:

• Fig. 18 provides the load-displacement curves for mortar mix 1 at 7 days.
• Fig. 19 provides the load-displacement curves for mortar mix 1 at 14 days.
• Fig. 20 provides the load-displacement curves for mortar mix 1 at 28 days.
• Fig. 21 provides the load-displacement curves for mortar mix 1 at 100 days.
• Fig. 22 provides the load-displacement curves for mortar mix 2 at 7 days.
• Fig. 23 provides the load-displacement curves for mortar mix 2 at 14 days.
• Fig. 24 provides the load-displacement curves for mortar mix 2 at 28 days.
• Fig. 25 provides the load-displacement curves for mortar mix 2 at 100 days.

## 2 Experimental materials and methodology

A series of testing was conducted on identifying a suitable test configuration for the PSP test. A number of tests were conducted to determine key physical aggregate properties. These included loose bulk density, oven dry particle density, apparent particle density, saturated surface dry (SSD) particle density and water absorption, and were carried out in accordance with I.S. EN 1097-3:1998 [2] and I.S. EN 1097-6:2013 [3]. The gradation for the fine aggregate and its physical properties used in the preparation of mortar samples can be found in [1]. Mortar samples of 150 mm cube were prepared by mixing the aggregate, cement and water in a mechanical mixer. The chosen cement was a commercially available CEM III/A produced to I.S. EN 197-1 [4]. The fine aggregate was composed of locally available concrete sand and crushed rock fines and were combined in a 2:1 ratio. The aggregates were used in an oven dry condition during the preparation of samples.

After curing, holes were drilled in the middle of one face of the cube sample using a pillar drill. Drilling was carried out in two steps to reduce geometric deviations of the drilled hole. A smaller drill bit was used to create a pilot hole, before utilising a larger bit to provide the desired diameter of the drill hole. The hole was then cleaned by blowing air at high pressure. The screw was inserted in the drilled hole by applying a controlled torque using a Hilti SIW22T-A impact wrench.

To test the sample, a quasi-static tensile action was applied to pull the screw from the sample using an Instron 1274 servo-hydraulic frame. The sample was held within a purpose-designed steel frame; this consisted of top and bottom plates held together with 8 threaded bars. The bottom plate featured a central hole through which the screw protrudes. The top plate was held in a fixed position by the upper jaw of the Instron while a tensile load is applied to the screw at a constant displacement rate of 0.10 mm/sec, pulling it from the sample. The load and displacement were continuously measured and recorded at a sampling rate of 100 Hz. The difference between the displacement of the screw head and the sample surface were measured using LVDTs in order to accurately determine the actual displacement of the screw relative to the concrete. The obtained load and displacement data was then converted to a load-displacement curve using Excel 2013. Other data of interest was obtained from the raw data such as peak load and its location.

## 3 Testing programme

The experimental programme consisted of three phases. The first phase includes preliminary investigation to confirm the consistency of the failure pattern in concrete during the proposed PSP test. by assessing the influence of presence of reaction ring around the screw and mode of failure. Based on this testing a set-up involving the reaction ring was accepted and key geometric parameters were identified for the next phase of the work.

The next phase was designed to obtain an appropriate and robust test configuration in mortar, where parameters such as screw diameter, diameter and depth of drilled hole and compressive strength of mortar were used. Arising from this testing, a number of decisions were made based on a qualitative assessment of the data; this discussion is available in the paper by [1] and led to the selection of a chosen test configuration.

The final phase was to ascertain the influence of compressive strength of mortar on the pullout load for a selected configuration (from 2nd phase) of the PSP test. Two mortar mixes were designed and each mix was tested at 7, 14, 28 and 100 days. A test configuration selected from the previous phase of the research was chosen for this phase of the research. Four PSP tests were carried out for each strength level and an average was considered for further analysis. Mortar compressive strength was obtained from the standard compressive strength test of three 100 mm cube samples. In total, 32 PSP tests and 24 compression tests were carried out in the final stage of testing

## Declaration of Competing Interest

The authors declare that they have no known competing financial interests or personal relationships which have, or could be perceived to have, influenced the work reported in this article.

## Appendix Supplementary materials

Fig. 1: Load-displacement curves for test set up 1. Fig. 2: Load-displacement curves for test set up 2. Fig. 3: Load-displacement curves for test set up 3. Fig. 4: Load-displacement curves for test set up 4. Fig. 5: Load-displacement curves for test set up 5. Fig. 6: Load-displacement curves for test set up 6. Fig. 7: Load-displacement curves for test set up 7. Fig. 8: Load-displacement curves for test set up 8. Fig. 9: Load-displacement curves for test set up 9. Fig. 10: Load-displacement curves for test set up 10. Fig. 11: Load-displacement curves for test set up 11. Fig. 12: Load-displacement curves for test set up 12. Fig. 13: Load-displacement curves for test set up 13. Fig. 14: Load-displacement curves for test set up 14. Fig. 15: Load-displacement curves for test set up 15. Fig. 16: Load-displacement curves for test set up 16. Fig. 17: Load-displacement curves for test set up 17. Fig. 18: Load-displacement curves for Mix 1 at 7 days. Fig. 19: Load-displacement curves for Mix 1 at 14 days. Fig. 20: Load-displacement curves for Mix 1 at 28 days. Fig. 21: Load-displacement curves for Mix 1 at 100 days. Fig. 22: Load-displacement curves for Mix 2 at 7 days. Fig. 23: Load-displacement curves for Mix 2 at 14 days. Fig. 24: Load-displacement curves for Mix 2 at 28 days. Fig. 25: Load-displacement curves for Mix 2 at 100 days. Image, application 1
